# Plitidepsin Has a Safe Cardiac Profile: A Comprehensive Analysis

**DOI:** 10.3390/md9061007

**Published:** 2011-06-09

**Authors:** Arturo Soto-Matos, Sergio Szyldergemajn, Sonia Extremera, Bernardo Miguel-Lillo, Vicente Alfaro, Cinthya Coronado, Pilar Lardelli, Elena Roy, Claudia Silvia Corrado, Carmen Kahatt

**Affiliations:** Clinical Oncology, Pharma Mar S.A., Colmenar Viejo, Madrid 28770, Spain; E-Mails: asoto@pharmamar.com (A.S.-M.); sszyldergemajn@pharmamar.com (S.S.); sextremera@pharmamar.com (S.E.); bdemiguel@pharmamar.com (B.M.-L.); ccoronado@pharmamar.com (C.C.); plardelli@pharmamar.com (P.L.); eroy@pharmamar.com (E.R.); cscorrado@pharmamar.com (C.S.C.); ckahatt@pharmamar.com (C.K.)

**Keywords:** plitidepsin, cardiac toxicity, single agent, chemotherapy, cancer

## Abstract

Plitidepsin is a cyclic depsipeptide of marine origin in clinical development in cancer patients. Previously, some depsipeptides have been linked to increased cardiac toxicity. Clinical databases were searched for cardiac adverse events (CAEs) that occurred in clinical trials with the single-agent plitidepsin. Demographic, clinical and pharmacological variables were explored by univariate and multivariate logistic regression analysis. Forty-six of 578 treated patients (8.0%) had at least one CAE (11 patients (1.9%) with plitidepsin-related CAEs), none with fatal outcome as a direct consequence. The more frequent CAEs were rhythm abnormalities (*n* = 31; 5.4%), mostly atrial fibrillation/flutter (*n* = 15; 2.6%). Of note, life-threatening ventricular arrhythmias did not occur. Myocardial injury events (*n* = 17; 3.0%) included possible ischemic-related and non-ischemic events. Other events (miscellaneous, *n* = 6; 1.0%) were not related to plitidepsin. Significant associations were found with prostate or pancreas cancer primary diagnosis (*p* = 0.0017), known baseline cardiac risk factors (*p* = 0.0072), myalgia present at baseline (*p* = 0.0140), hemoglobin levels lower than 10 g/dL (*p* = 0.0208) and grade ≥2 hypokalemia (*p* = 0.0095). Treatment-related variables (plitidepsin dose, number of cycles, schedule and/or total cumulative dose) were not associated. Electrocardiograms performed before and after plitidepsin administration (*n* = 136) detected no relevant effect on QTc interval. None of the pharmacokinetic parameters analyzed had a significant impact on the probability of developing a CAE. In conclusion, the most frequent CAE type was atrial fibrillation/atrial flutter, although its frequency was not different to that reported in the age-matched healthy population, while other CAEs types were rare. No dose-cumulative pattern was observed, and no treatment-related variables were associated with CAEs. Relevant risk factors identified were related to the patient’s condition and/or to disease-related characteristics rather than to drug exposure. Therefore, the current analysis supports a safe cardiac risk profile for single-agent plitidepsin in cancer patients.

## 1. Introduction

The use of multimodality treatment, including surgery, chemotherapy, radiotherapy, and targeted therapies has significantly decreased cancer-related mortality. As more oncological patients now have a longer life expectancy, treatment-related comorbidity and its prevention has become an important issue in cancer treatment. Cardiac disease in cancer patients is common and can be due to the malignancy itself, co-morbidities like age or pre-existing heart disease, or cardiotoxic chemotherapeutic agents. In fact, cardiac toxicity is one of the most worrisome side effects of anticancer therapy because the gain in life expectancy obtained with chemotherapy might be counter-balanced by a detrimental effect in the quality of life of the patients, or even lead to an increased mortality due to cardiac problems. Several well-established and widely used anticancer agents have been associated with an increased risk of cardiac toxicity: Anthracyclines, fluoropyrimidines, etoposide, high-dose alkylating agents, interferon, interleukin-2, taxanes, monoclonal antibodies (trastuzumab, bevacizumab) and tyrosine kinase inhibitors (sunitinib, sorafenib) among others [1,2]. Nevertheless, their mechanisms (known or unknown) of inducing cardiac toxicity are different.

The most notable chemotherapy-related cardiac toxicity is myocardial damage, which may lead to impaired cardiac function and overt congestive heart failure. This type of cardiotoxicity has been observed in patients treated with anthracyclines or high doses of alkylating agents, and is known as type I chemotherapy-related cardiac dysfunction (*i.e*., it is present from the earliest administration of the drug) [3]. Type II chemotherapy-related cardiac dysfunction, characterized by reversibility and lack of dependence on dose or re-exposure to the agent, is not associated with myocyte damage and has been observed with the administration of trastuzumab or alemtuzumab [4–6]. Other antineoplastic agents (anthracyclines, 5-fluorouracil, some platinum compounds, multitargeted tyrosine-kinase inhibitors, anti HER-2, anti-vascular endothelial growth factor (VEGF), vascular disruption agents and histone deacetylase inhibitors (HDACIs)) may affect QT interval duration in the electrocardiogram [7]. This effect is also observed with other different agents that are widely used among cancer patients, such as concomitant medications to reduce the side effects of chemotherapy, particularly, some serotonin receptor antagonist antiemetic agents [8]. Fortunately, cardiac side effects remain uncommon with most chemotherapeutic agents, and several preventive or protective strategies are currently available [9]. Cardiac toxicity resulting in myocardial dysfunction can become apparent immediately or long after the end of therapy, and often is irreversible. Therefore, early and accurate detection of cardiac injury is crucial because it can lead to early therapeutic interventions. Close and accurate monitoring of cardiac function is important for early detection of cardiac dysfunction during clinical development of new anticancer therapies, which might lead to timely-appropriate preventive measures.

Aplidin^®^ (plitidepsin) is a cyclic depsipeptide originally isolated from the Mediterranean tunicate *Aplidium albicans* and currently produced by chemical synthesis [10]. Plitidepsin has been proven to be active against several human malignant cell lines both *in vitro* and *in vivo* at nanomolar concentration ranges [11]. The primary mode of action of plitidepsin has not been fully elucidated, though evidence available suggests that plitidepsin induces oxidative stress, which increases levels of cell membrane phospholipid and DNA oxidation [12], decreases intracellular levels of glutathione and activates the Rac1-JNK pathway, thereby resulting in both caspase-dependent and -independent cellular apoptosis [13–15]. In several preclinical models, plitidepsin has also been shown to have antiangiogenic properties, mainly characterized by inhibition of the expression of different angiogenic genes, including VEGF and its receptor (VEGFR-1) [16–19]. Plitidepsin has been extensively studied as single-agent chemotherapy in phase I and II clinical trials [20–30], and clinically relevant antitumor activity has been observed in a wide-spectrum of solid as well as hematological malignancies such as malignant melanoma [25], multiple myeloma [29], peripheral T-cell lymphoma [30] and renal cell carcinoma [23]. Plitidepsin is currently being explored in a pivotal randomized phase III trial (ADMYRE study) conducted in patients with relapsed/refractory multiple myeloma who have failed standard available therapies.

Plitidepsin has neither shown cardiac toxicity during preclinical/toxicology studies nor has been reported in early phase I clinical trials [20–22]. However, other chemically-related compounds such as depsipeptides under clinical evaluation (e.g., FK228, and HDACI) have shown QTc prolongation, moderate ventricular tachycardia and sudden death attributed to possible fatal ventricular arrhythmia in phase II clinical trials [31,32]. Although cardiotoxic effects of FK228 have already been identified in preclinical toxicology studies in dog [31], its ability to induce cardiac toxicity appears to be heterogeneous and may vary based on the patient population [33]. Electrocardiogram (ECG) abnormalities may be a class effect, as this has been reported with other HDACI inhibitors such as LBH589 or LAQ824 [33].

As other cyclic depsipeptides have been linked to increased cardiac toxicity, the current analysis evaluated the cardiac safety profile of plitidepsin based on the safety information on cardiac adverse events (CAEs) that occurred during clinical trials to evaluate plitidepsin as single-agent chemotherapy in adult patients with advanced solid tumors or hematological malignancies.

## 2. Patients and Methods

Clinical trials included in this cardiac safety analysis were 4 phase I and 14 phase II studies evaluating plitidepsin as single-agent chemotherapy in a total of 578 adult patients (see details in Table 1). All available information on CAEs was retrieved from Pharma Mar’s internal Pharmacovigilance and Clinical Trials databases. The reviewed sources of information were Case Report Forms (CRFs), serious adverse event (SAE) reports, follow-up reports and supplementary reports attached or requested. Median follow-up was 12.0 months (95% CI, 7.6–13.8) for phase I trials and 12.3 months for phase II trials (95% CI, 11.0–13.7). Cutoff for data analysis was 20 November 2008.

All adverse events (AEs) with preferred terms of the Medical Dictionary for Regulatory Activities (MedDRA) that could be potentially classified as cardiac were evaluated. Additionally, events included in other systems as well in those patients with any vascular risk associated (prior history or within the trial) were also reviewed. Laboratory data were also reviewed in those cases with grade 3/4 creatine phosphokinase increase and/or troponin (troponin C or troponin I) elevations. Patients’ prior history and baseline information were searched for any associated vascular risk (diabetes, hypercholesterolemia, hypertension, smoking, prior myocardial infarction or angina, *etc.*). All events retrieved using the aforementioned criteria were reviewed to identify and exclude from analysis potential CAEs due to obvious cancer-related symptoms (e.g., sinusal tachycardia in anemic patients, chest pain obviously related to tumor involvement of the chest). CAEs were divided into three groups: “rhythm abnormalities” (including regular and irregular supraventricular thacychardia), “myocardial injury” (including possible ischemic-related events and other myocardial events, such as cardiac failure, cardiomegaly, cardiomyopathy, ejection fraction decrease, *etc.*), and “miscellaneous” (CAEs that did not fit into any of the other two categories). The information obtained was tabulated and a descriptive analysis is reported here.

To avoid potential bias, candidate variables were explored by univariate analysis regardless of the relationship attributed to the CAE with plitidepsin by the investigators (related, unrelated or unknown). Univariate logistic regression was used to study the relationship between the occurrence of a CAE and categorical and continuous patient and disease characteristics, and other observed toxicities or laboratory abnormalities. Those variables found to be clinically relevant and/or statistically significant (or of borderline significance) in this analysis were further assessed as covariates in a multivariate analysis using logistic regression with stepwise variable selection and multiple correspondence analysis methods.

Selected ECG variables (Bazett’s corrected QT interval (QTc) and heart rate (HR)) were analyzed in patients with paired ECG data (*i.e*., available before and after plitidepsin infusion, *n* = 136).

Pharmacokinetic (PK) data available from individual clinical trials (four phase I studies and nine phase II studies; see details in Table 1) and/or simulated data using population PK analyses were correlated with the occurrence of CAEs. In the individual studies, doses ranged from 0.13 to 8.00 mg/m^2^, and were administered as 1 h or 24 h infusion weekly, 3 h or 24 h infusion bi-weekly, or 1 h infusion for 5 consecutive days every 3 weeks. To evaluate the potential relationship between blood plitidepsin concentrations and QTc and HR values, a total of 90 patients who had ECGs performed during treatment with plitidepsin and PK data during first infusion were analyzed. The plitidepsin concentration at the time when the ECG had been obtained was calculated using the whole blood plitidepsin concentration at the ECG times in which they were simulated taking the individual Bayesian PK parameters of the final population PK model and considering the real treatment history of each patient. In total, 411 patients and 5585 plitidepsin concentration measurements (including 2900 blood and 2685 plasma concentrations) were pooled in the population PK analysis. An open, 3-compartment disposition model with linear elimination and linear distribution from the central compartment to peripheral compartments was used to describe the PK of plitidepsin in plasma. Between and within subject variabilities were assumed to be log-normally distributed. PK exposure parameters (maximum concentration in plasma (*C*_max_) and area under the curve (AUC) from day 0 to day 28) and other dose-independent PK parameters (clearance, half-life, *etc.*) were used to evaluate the relationship between patient PK characteristics and the appearance of CAEs. For the statistical analysis of the relationship between the exposure parameters and the development or not of a CAE, a logistic regression model was used to analyze the incidence of the event. To avoid loss of cases with respect to other covariates included in this multivariate analysis, the PK parameters of the 167 patients that did not have PK data were calculated and included using the real dosing and the typical PK population parameters [35].

## 3. Results

Forty-six of the 578 patients (8.0%) treated with plitidepsin as single agent had at least one CAE (Table 2). The majority of these CAEs (37 of 46 patients) occurred in phase II trials, particularly in two studies evaluating plitidepsin in patients with pancreatic and prostate cancer. Eleven patients (1.9%) had 15 CAEs related to plitidepsin. These CAEs consisted of palpitations (*n* = 4), chest pain (*n* = 2), and supraventricular tachycardia, tachycardia, atrial fibrillation, atrial flutter, cardiac failure, acute cardiomyopathy, change in ECG, ECG QT prolonged, and ejection fraction decreased (*n* = 1 each). None of the patients who experienced a CAE (whether related, unrelated or unknown) had a fatal outcome as a direct consequence.

Classification of CAEs is shown in Table 3. The most frequent type was rhythm abnormalities (*n* = 31; 5.4%), with irregular supraventricular tachycardia (*n* = 15; 2.6%) being the most common (atrial fibrillation/flutter accounting for most cases). Univariate analysis showed that patients without known cardiac risk factors or relevant cardiac concomitant medication at baseline, lower performance status, absence of significant myalgia at baseline, normal or low body mass index, and higher hemoglobin levels during treatment were at a significantly lower risk of experiencing rhythm abnormalities. Remarkably, no treatment-related exposure variables were significant in predicting occurrence of these events.

Myocardial injury events were relatively rare (*n* = 17; 3.0%). Chest pain (*n* = 4; 0.7%), myocardial ischemia (*n* = 3; 0.5%) and cardiac failure (*n* = 3; 0.5%) were the most common. As mentioned above, none of these CAEs had fatal outcome as a direct consequence. Univariate analysis showed patient-dependant and disease-dependant variables, such as older age (no events occurred in patients under 45 years), prostate or pancreatic cancer primary diagnosis, higher bilirubin levels during treatment, lower creatinine clearance during treatment, and at least grade 2 hypokalemia during treatment) as significantly correlated with the occurrence of myocardial injury events.

The miscellaneous category (*n* = 6; 1.0%) included all other CAEs. The most reported were pericardial effusion (*n* = 3; 0.5%) and hypotension (*n* = 2; 0.3%). None of these events was recorded as related to plitidepsin in the databases. Patients with hemoglobin values lower than 10 g/dL during treatment, and those with prior history of mediastinal radiotherapy, were significantly more likely to experience events grouped in this category.

Multivariate analysis showed 5 significant variables significantly associated with CAEs: Prostate or pancreatic cancer primary diagnosis (*p* = 0.0017; odds ratio (OR) = 4.217); known baseline cardiac risk factors (*p* = 0.0072; OR = 3.034); grade ≥2 hypokalemia during treatment (*p* = 0.0095; OR = 3.851); myalgia present at baseline (*p* = 0.0140; OR = 5.015), and low hemoglobin values (<10 g/dL) during treatment (*p* = 0.0208; OR = 2.195) (Table 4). Of note, all these five variables are patient-related or disease-related characteristics.

**Figure 1:**
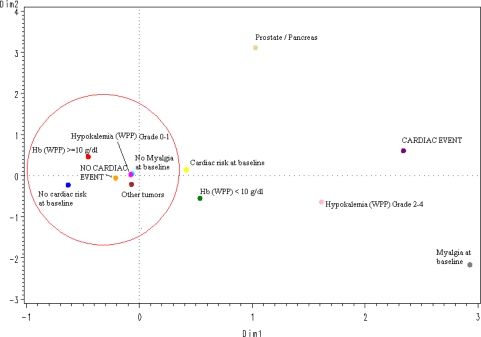
Multiple correspondence analysis (overall); Hb: Hemoglobin; WPP: worst per patient.

The spatial distribution according to a multiple correspondence analysis of the values of the 5 significant variables selected in the logistic multivariate regression model (Figure 1) predicted the absence of CAEs in an accurate way: All protective categories (tumors different than prostate or pancreatic cancer; grade 0–1 hypokalemia, no myalgia at baseline, no cardiac risk or relevant concomitant medication at baseline, and hemoglobin ≥10 g/dL) were grouped around the “no-CAE” area.

Electrocardiograms performed before and after plitidepsin administration showed no relevant effect on QTc interval and HR (Table 5). The mean (SD) increase in QTc values was 2.51 (32.99) msec in all patients with available ECG (*n* = 136), and 4.85 (31.66) msec in patients with a CAE (*n* = 20). The mean (SD) increase in HR values was 3.39 (9.92) bpm in all patients and 6.05 (9.76) bpm in patients with a CAE.

**Figure 2:**
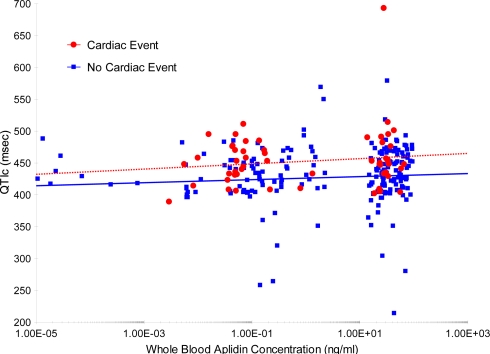
Simulated whole blood plitidepsin concentration *vs.* Bazett’s corrected QT interval (QTc) interval (left graph) and heart rate (right graph) for patients without and with a cardiac event. The whole blood plitidepsin concentration at the ECG times was simulated taking the individual post-hoc PK parameters of population PK model and considering the real treatment history of each patient. Lines are the regression lines for patients without (blue line) and with a cardiac event (red line). ECG: electrocardiogram; PK: pharmacokinetic.

An additional analysis was performed to evaluate a potential relationship between whole blood plitidepsin concentrations and QTc. For this evaluation, all ECGs performed during treatment with plitidepsin were considered (total of 263 ECGs), and not only those ECGs with a pair before/after each infusion (Figure 2). No apparent relationship was found between plitidepsin concentrations and QTc interval.

PK exposure parameters (*C*_max_ and AUC_0–28day_) were used to evaluate the relationship between patient PK characteristics and the appearance of CAEs. Patients without CAE and with PK data (*n* = 375) had a whole blood *C*_max_ and AUC_0–28day_ of 58.8 ng/mL and 3374 h·ng/mL, respectively, while those with a CAE (*n* = 36) had a whole blood *C*_max_ and AUC_0–28day_ of 61.0 ng/mL and 3236 h·ng/mL, respectively (Table 6).

The results of the logistic regression analyses are shown in Table 7. The ORs for all analyses were very close to 1; this indicates that the contribution of the evaluated PK parameters to the probability of developing a CAE was very low. None of these comparisons was statistically significant.

## 4. Discussion

Plitidepsin, a cyclic depsipeptide of marine origin, is an investigational drug currently in clinical development as a single agent as well as in combination with other anticancer agents for the treatment of different solid tumors and hematological malignancies. Plitidepsin did not show a cardiotoxic profile in preclinical/toxicology studies, which involved both *in vitro* studies (HERG assay and study using Purkinje fibers) and an *in vivo* study in the dog, as well as a exploratory study of the cardiac effects of escalating doses of plitidepsin in a guinea pig Langendorff preparation [36]. CAEs in plitidepsin trials were relatively rare (affecting 8% of the patients) and the different categories of CAEs evaluated had frequencies lower than 3%; therefore, they are unlikely to share a common pathogenesis and/or common predisposing factors (e.g., atrial fibrillation and myocardiopathy or pericardial effusion). The current comprehensive safety analysis shows that CAEs occurred in plitidepsin trials with a median follow-up of about one year were clinically heterogeneous, with the most frequent CAE type being non-life threatening rhythm alterations (mostly atrial fibrillation/flutter). It should be noted that the frequency of these rhythm abnormalities (5.4%) was not different to what is actually reported in the age-matched healthy population [37–39].

Rhythm abnormalities appeared to be characterized by random onset during treatment and were usually reversible even without treatment discontinuation. Relevant predisposing factors identified in univariate and multivariate analyses were mostly related with patient’s baseline characteristics and disease-related characteristics rather than with drug exposure or treatment-related characteristics. Rhythm abnormalities occurred more frequently in patients with predisposing cardiac factors, with lower hemoglobin values than 10 g/dL at some point during treatment and poor performance status at baseline. The higher incidence observed in phase II studies (6.9% *vs.* 5.4% in all studies) may be explained by the introduction of systematic screening measures in all phase II studies since 2006, with extensive serial ECGs evaluations before and after plitidepsin infusions that could detect events that otherwise would have been missed. This higher incidence may have also been caused by two individual phase II studies in specific solid tumor types (prostate and pancreatic cancer) that represent a particularly frail and susceptible subpopulation of patients. Remarkably, potentially life-threatening arrhythmias (e.g., ventricular arrhythmias) did not occur within plitidepsin trials.

Events of the myocardial injury type (whether ischemic or not) were relatively rare and frequency in phase II studies was not increased significantly compared to phase I studies despite the extensive monitoring measures implemented. Of note, none of these CAEs had a fatal outcome as a direct consequence. Known increased cardiac risk conditions as well as prior anthracycline treatment were not correlated with the occurrence of myocardial injury events; however, more restrictive inclusion criteria and better patient selection regarding these factors in all plitidepsin trials since 2006 may have had a role in preventing a rising of these events in phase II studies. Age, but not gender, was associated with an increased frequency of myocardial injury events. Patients with prostate and pancreatic cancer were significantly more likely to experience these events. Lower creatinine clearance was also associated with an increased cardiac risk. This alteration is commonly observed in prostate [40,41] and pancreatic cancer patients [42], as well as an overall increased risk of thromboembolic events. Particularly remarkable with respect to potential associations between musculoskeletal and cardiac toxicity was the lack of a significant association in univariate analyses between relevant musculoskeletal related symptoms (*n* = 332) that plitidepsin is well known to cause (creatine phosphokinase increased at baseline or during treatment, muscular weakness, muscle cramps and fatigue) and the occurrence of CAEs. Thus, patients who experienced musculoskeletal toxicity while on plitidepsin treatment did not show an increased risk of myocardial injury.

Some laboratory alterations found during treatment (hemoglobin decreases or grade ≥2 hypokalemia) were statistically significant in the multivariate analyses. No laboratory alterations at baseline were significantly associated with an increased risk of CAEs, but this is an expected finding as study inclusion criteria generally excluded patients with extreme laboratory values. Cancer patients with anemia are more prone to have arrhythmias, which may trigger the development of further CAEs, such as ischemia, or may even be the direct cause of events such as palpitations [43]. Hypokalemia is known to be associated with several arrhythmias and QT prolongation [44], but is not commonly associated to plitidepsin treatment. The associations with other metabolic disturbances known to cause arrhythmias (e.g., hypocalcemia or hyponatremia) were non-significant. Of note, none of the severe arrhythmias more commonly associated with metabolic disturbances (e.g., Torsades de pointes, ventricular arrhythmias and severe QT prolongation) occurred in patients treated with plitidepsin.

The incidence of cardiotoxicity related to oncological therapy depends on treatment-related factors (type of drug, cumulative dose and schedule of administration, combination of potentially cardiotoxic drugs, or association with mediastinal radiotherapy), but also on patient-related factors (age, presence of cardiovascular risk factors or coexisting cardiac disease, previous mediastinal irradiation, *etc.*) [45]. Some demographic variables are known to be associated with an increased cardiac risk of cardiac disease in the general population. The incidence of cardiac disease increases with age, and so does the incidence of cancer. The only significant relationship between age and CAEs was found for myocardial injury events where, remarkably, no such events occurred in patients younger than 45 years.

The results of multivariate analysis, after selecting the most significant, reproducible and representative variables found in univariate analyses, were consistent with those of univariate analyses, although, as expected given the relatively low overall incidence of CAEs, models were more representative for predicting patients at very low risk of experiencing these events than for predicting patients likely to experience them. The spatial distribution, according to a multiple correspondence analysis, of the five variables found significant in the multivariate analysis accurately predicted the absence of CAEs in patients with a cancer diagnosis other than prostate or pancreatic cancer, without myalgia at baseline, without known cardiac risks at baseline, with less than grade 2 hypokalemia during treatment, and with hemoglobin levels higher than 10 g/dL during treatment. Notably, none of these significant variables was related with plitidepsin treatment exposure; this finding is consistent with the clinical and the PK analyses. However, a definite conclusion cannot be drawn yet due to the relatively small number of patients treated and the relatively low incidence of CAEs observed to date. Furthermore, other factors that may have been unnoticed in this analysis might also play a role in the occurrence of CAEs.

A slight mean increase (2.51 ms) was found in the QTc interval after plitidepsin infusion compared with the value obtained before the infusion. Plitidepsin concentration did not appear to have an effect on the magnitude of the QTc interval. Nevertheless, the analysis presented here has some important flaws: lack of uniformity in the method for measuring QT intervals, absence of a control for the circadian rhythm, and presence of premedication (antiemetics) in all patients known to potentially affect QT interval [8]. Therefore, this analysis should be considered an exploratory approach for the evaluation of any potential effect of plitidepsin on the QT interval, where no alarming signs have been detected so far and no definite conclusions can be drawn.

*C*_max_ and AUC_0–28day_ were used to evaluate the relationship between patient PK characteristics and appearance of CAEs. Based on the real plasma and whole blood concentrations, a population PK model was developed to derive the individual *post-hoc* PK parameters. A whole blood PK profile from day 0 to day 28 was simulated taking into account the derived individual post-hoc PK parameters and the true treatment history of each patient. This was considered the best way to define a relevant PK exposure with the integration of all patients into one analysis. The difficulties encountered were the availability of plasma and whole blood concentrations in different patients (some only had whole blood concentrations, others only had plasma concentrations and some had both) and the very different schedules of administration used in the clinical trials. The doses given to patients included in the phase II studies were those defined as the recommended dose for every schedule. Thus, in the evaluation of these patients, the dose-independent parameters (clearance, volume of distribution at steady-state and half-life) were also taken into consideration. None of the PK parameters evaluated had a significant impact on the probability of developing a CAE, either when all events were analyzed together or when the analysis was performed in the different categories for each CAE.

## 5. Conclusion

The CAEs observed in plitidepsin trials are clinically heterogeneous. The most frequent CAE was atrial fibrillation/atrial flutter, although its incidence was not different to that reported in the age-matched healthy population. Other CAE types were rare. Relevant predisposing factors identified in univariate and multivariate analyses were mostly related with patient’s baseline characteristics and disease-related characteristics rather than with drug exposure or treatment-related characteristics, and none of the explored PK parameters showed a correlation. No dose-cumulative pattern was observed, and no treatment-related variables were associated with CAEs. Therefore, the current analysis with data available on 578 adult advanced cancer patients treated with single-agent plitidepsin supports a safe cardiac risk profile for this agent. However, although cardiac safety does not seem to be of special concern with the available data, comprehensive monitoring measures remain in place and are operative in plitidepsin trials to identify any potential cardiac safety risk as early as possible in order to prompt and adequately manage any early sign in patients exposed to plitidepsin. Preventive measures in ongoing trials with plitidepsin include inclusion of patients with adequate hemoglobin levels at baseline; enrolment of patients with prior exposure to anthracyclines up to a maximal cumulative doxorubicin-equivalent total dose of 450 mg/m^2^; extension of follow-up for LVEF assessments; monitoring of ECG during treatment, and assessment of chronic toxicity. To date, measurement of troponin in asymptomatic patients has not proven very useful to identify the cardiovascular risk in trials with plitidepsin. All clinical trials included in this analysis had no control arm. A control arm without plitidepsin treatment might be the best way to prospectively assess the incidence of CAEs, as this may compensate for known and unknown factors present in cancer patients, including concomitant treatments, *etc.* Currently, a phase III randomized trial (ADMYRE study) is ongoing and evaluating plitidepsin in combination with dexamethasone *versus* dexamethasone alone in patients with relapsed/refractory multiple myeloma. This trial will also help to evaluate the effect of adding plitidepsin on cardiac events with respect to dexamethasone monotherapy in this frail patient population, as cardiac safety is intensively and symmetrically monitored in both arms, as well to obtain information on the usefulness of troponin assessment in asymptomatic patients.

## Figures and Tables

**Table 1:** Cardiac monitoring and pharmacokinetic data available in clinical trials included in this cardiac safety analysis (*n* = 18 studies).

			**Pts**	**Prospective cardiac monitoring**						
**Study**	**Phase**	**Main indication**		**Troponin**	**LVEF**	**CPK-MB**	**ECG monitoring**	**Intensive ECGs monitoring**	**PK data**	**Ref.**
1	I	Solid tumors or lymphomas	62	Yes	No	No	Yes	No	Yes	[34]
2	I	Solid tumors or lymphomas	48	No	No	Yes	Yes	No	Yes	[22]
3	I	Solid tumors or lymphomas	67	No	No	No	Yes	No	Yes	[20]
4	I	Solid tumors or lymphomas	37	No	No	No	Yes	No	Yes	[21]
5	II	Renal or colorectal cancer	81	No	No	Yes	No	No	Yes	[23]
6	II	Medullary thyroid cancer	16	No	Yes	No	Yes	No	Yes	[27]
7	II	Pancreatic cancer	19	No	Yes	No	Yes	No	No	NA
8	II	NSCLC	21	No	No	No	Yes	No	No	[28]
9	II	Urothelial cancer	21	No	Yes	Yes	Yes	No	Yes	[26]
10	II	SCLC	19	No	Yes	Yes	Yes	No	Yes	[24]
11	II	Second-line melanoma	37	No	Yes	Yes	Yes	No	Yes	[25]
12	II	Head and neck cancer	10	No	No	Yes	Yes	No	No	NA
13	II	Refractory androgen-independent prostate cancer	8	Yes	Yes	Yes	Yes	Yes	Yes	NA
14	II	Indolent Non-Hodgkin’s lymphoma	8	Yes	Yes	Yes	Yes	No	No	NA
15	II	Aggressive non-Hodgkin’s lymphoma	48	Yes	Yes	Yes	Yes	Yes	Yes	[30]
16	II	Multiple myeloma	51	Yes	Yes	Yes	Yes	Yes	Yes	[29]
17	II	Acute lymphoblastic leukemia	17	Yes	Yes	Yes	Yes	No	Yes	NA
18	II	First-line melanoma	8	Yes	Yes	Yes	Yes	Yes	No	NA

		**Total (%)**	**578**	**7 (38.9%)**	**11 (61.1%)**	**12 (66.7%)**	**17 (94.4%)**	**4 (23.5%)**	**13 (72.2%)**	

CPK-MB: creatine phosphokinase-MB fraction; ECG: electrocardiogram; LVEF: left ventricular ejection fraction; NA: not available; NSCLC: non-small cell lung cancer; PK: pharmacokinetic; Pts: patients; SCLC: small cell lung cancer.

**Table 2:** Cardiac adverse events reported with plitidepsin treatment.

	**Patients treated**	**Patients with cardiac event (regardless of relationship)**	**Patients with cardiac event related to plitidepsin**	Patients with cardiac event of unknown causality *
Total phase I	214	9 (4.2%)	2 (0.9%)	2 (0.9%)
Total phase II	364	37 (10.2%)	9 (2.5%)	14 (3.8%)
**Total**	**578**	**46 (8.0%)**	**11 (1.9%)**	**16 (2.8%)**

Data shown are *n* (%) of patients; A patient may have more than one cardiac adverse event (CAE); The median number of plitidepsin-related CAEs was 1 (range 1–2), and the median number of CAEs (regardless of relationship) was 1 (range 1–3). Four patients had ≥2 plitidepsin-related CAEs; 14 patients had ≥2 CAEs (regardless of relationship);

* Relationship with plitidepsin unknown.

**Table 3:** Cardiac adverse events (regardless of relationship) reported with plitidepsin treatment.

	**All studies (*n*** = **578)**	**Phase II studies (*n*** = **364)**

Group/Subgroup/Terms *	**Patients (%)**	**Patients (%)**
**Rhythm Abnormalities**	**31 (5.4%)**	**25 (6.9%)**
**Regular Supraventricular Tachycardia**	**6 (1.0%)**	**1 (0.3%)**
Sinus tachycardia	1 (0.2%)	1 (0.3%)
Supraventricular arrhythmia	1 (0.2%)	0 (0.0%)
Supraventricular tachycardia	1 (0.2%)	0 (0.0%)
Tachycardia	3 (0.5%)	0 (0.0%)
**Irregular Supraventricular Tachycardia**	**15 (2.6%)**	**14 (3.8%)**
Atrial fibrillation	11 (1.9%)	10 (2.7%)
Atrial flutter	5 (0.9%)	5 (1.4%)
Heart rate irregular	1 (0.2%)	1 (0.3%)
**Other Rhythm Abnormalities**	**13 (2.2%)**	**13 (3.6%)**
Arrhythmia	1 (0.2%)	1 (0.3%)
Atrioventricular block	1 (0.2%)	1 (0.3%)
Change in electrocardiogram	1 (0.2%)	1 (0.3%)
Electrocardiogram QT prolonged	5 (0.9%)	5 (1.4%)
Palpitations	6 (1.0%)	6 (1.6%)

**Myocardial Injury**	**17 (3.0%)**	**14 (3.8%)**
**Possible Ischemic Related**	**10 (1.7%)**	**8 (2.2%)**
Acute myocardial infarction	1 (0.2%)	1 (0.3%)
Cardiac troponin I increased	1 (0.2%)	1 (0.3%)
Chest pain	4 (0.7%)	2 (0.5%)
Electrocardiogram ST-T change	1 (0.2%)	1 (0.3%)
Myocardial ischemia	3 (0.5%)	3 (0.8%)
**Other Myocardial Injury**	**7 (1.2%)**	**6 (1.6%)**
Cardiac failure	3 (0.5%)	2 (0.5%)
Cardiomegaly	1 (0.2%)	1 (0.3%)
Cardiomyopathy	2 (0.3%)	2 (0.5%)
Ejection fraction decreased	1 (0.2%)	1 (0.3%)

**Miscellaneous Cardiac Adverse Events**	**6 (1.0%)**	**3 (0.8%)**
Cardiac amyloidosis	1 (0.2%)	1 (0.3%)
Hypertension	1 (0.2%)	0 (0.0%)
Hypotension	2 (0.3%)	2 (0.5%)
Pericardial effusion	3 (0.5%)	1 (0.3%)
Ventricular hypokinesia	1 (0.2%)	1 (0.3%)

**Total Adverse Events**	**46 (8.0%)**	**37 (10.2%)**

* A patient may have more than one cardiac adverse event (CAE). The median number of plitidepsin-related CAEs was 1 (range 1–2), and the median number of CAEs (regardless of relationship) was 1 (range 1–3). Four patients had ≥2 plitidepsin-related CAEs; 14 patients had ≥2 CAEs (regardless of relationship).

**Table 4:** Overall multivariate analysis of variables associated to cardiac adverse events with plitidepsin treatment.

Parameter *	**Reference value**	**DF**	**Estimate**	**Standard error**	**Wald chi-square**	*p*-value **	**OR**	**95% CI**	
**Tumor type**	Prostate or pancreatic cancer	1	1.4392	0.4585	9.8519	0.0017	4.217	1.717	10.360
**Cardiac risk and relevant concomitant medication at baseline**	Presence	1	1.1100	0.4131	7.2192	0.0072	3.034	1.350	6.819
**Hypokalemia (worst per patient)**	Grade ≥ 2	1	1.3484	0.5200	6.7249	0.0095	3.851	1.390	10.671
**Myalgia (at baseline)**	Presence	1	1.6124	0.6564	6.0349	0.0140	5.015	1.385	18.154
**Hemoglobin (worst per patient)**	<10 g/dL	1	0.7861	0.3402	5.3406	0.0208	2.195	1.127	4.275

* Three variables (hemoglobin <10 g/dL, hypokalemia grade ≥2 and presence of cardiac risk/relevant concomitant medication at baseline) lost statistical significance when unrelated CAEs were excluded from the multivariate analysis, possibly due to the low number of events and to the added new category (related, unknown and no CAE). Nevertheless, overall results were similar, thus showing that causal relationship assigned to the events was not a determinant factor;

** Wald Chi Square test (ordered by decreasing significance); CAE: cardiac adverse event; CI: confidence intervals; DF: degrees of freedom; OR: odds ratio.

**Table 5:** Bazett’s corrected QT interval (QTc) and heart rate (HR) values of all patients evaluated and grouped by the presence and type of cardiac adverse event.

**Group (number of pairs)**	**QTc (msec) Pre-infusion**	**QTc (msec) Post-infusion**	**ΔQTc (msec)**	**HR (bpm) Pre-Infusion**	**HR (bpm) Post-Infusion**	**ΔHR (bpm)**
**All** (*n* = 136) *	426.77 (38.78)	429.28 (40.25)	2.51 (32.99)	79.07 (15.33)	82.46 (13.17)	3.39 (9.92)
**No cardiac event** (*n* = 116)	424.88 (40.06)	426.98 (41.18)	2.10 (33.33)	79.92 (15.53)	82.86 (13.53)	2.94 (9.91)
**Cardiac event** (*n* = 20)	437.75 (28.65)	442.60 (32.09)	4.85 (31.66)	74.05 (13.39)	80.10 (10.87)	6.05 (9.76)
**Rhythm abnormalities** (*n* = 18)	439.44 (29.79)	442.67 (33.79)	3.22 (32.89)	75.44 (13.39)	80.44 (11.30)	5.00 (9.70)
**Myocardial injury** (*n* = 4)	429.00 (29.15)	437.50 (15.20)	8.50 (21.79)	68.75 (10.34)	77.75 (12.55)	9.00 (9.90)

Data shown are mean (SD);

* Only ECGs performed within 4 h after the end of the infusion are included because ECGs performed later were onsidered to not reflect the real QTc increase potentially caused by plitidepsin. A total of 136 pairs of measurements were available, corresponding to the same number of plitidepsin infusions; Bpm: beats per minute; ECG: electrocardiogram; HR: heart rate; SD: standard deviation.

**Table 6:** Mean (SD) plitidepsin pharmacokinetic parameters by category of the cardiac adverse event.

**Group (number of patients)**	***C*_max_ (ng/mL)**	**AUC_0–28day_ (h**·**ng/mL)**
**No Cardiac adverse Event** (*n* = 375)	58.8 (38.8)	3374 (4336)
**Cardiac Adverse Event** (*n* = 36)	61.0 (27.9)	3236 (2955)
**Rhythm Abnormalities** (*n* = 25)	61.5 (29.8)	3435 (3372)
**Regular supraventricular tachycardia** (*n* = 6)	57.2 (23.1)	5277 (3997)
**Irregular supraventricular tachycardia** (*n* = 12)	63.7 (34.6)	3197 (3640)
**Other rhythm abnormalities** (*n* = 10)	56.8 (26.2)	2064 (1449)
**Myocardial Injury** (*n* = 11)	69.5 (28.2)	4077 (3653)
**Possible ischemic related** (*n* = 7)	72.2 (28.8)	3668 (1696)
**Other myocardial injury** (*n* = 4)	64.7 (30.6)	4791 (6136)
**Miscellaneous Cardiac Adverse Event** (*n* = 7)	54.5 (19.7)	2645 (1672)

AUC: area under the curve; *C*_max_: maximum plasma concentration.

**Table 7:** Results of the logistic regression analysis evaluating the effect of *C*_max_ and AUC_0–28day_ on the probability of having a cardiac adverse event.

**Group (number of patients)**	**PK parameter**	**Estimate (β)**	**SE**	**OR**	**95% CI**		*p* value *
**Cardiac Adverse Event** (*n* = 36)	*C*_max_	0.001480	0.004360	1.001	0.993	1.010	0.7346
	AUC_0–28day_	−0.000008	0.000045	1.000	1.000	1.000	0.8521
**Rhythm Abnormalities** (*n* = 25)	*C*_max_	0.001760	0.005030	1.002	0.992	1.012	0.7264
	AUC_0–28day_	0.000003	0.000047	1.000	1.000	1.000	0.9449
**Regular supraventricular tachycardia** (*n* = 6)	*C*_max_	−0.001090	0.0112	0.999	0.977	1.021	0.9219
	AUC_0–28day_	0.000049	0.000049	1.000	1.000	1.000	0.3153
**Irregular supraventricular tachycardia** (*n* = 12)	*C*_max_	0.002890	0.00662	1.003	0.990	1.016	0.6624
	AUC_0–28day_	−0.000010	0.000076	1.000	1.000	1.000	0.8889
**Other rhythm abnormalities** (*n* = 10)	*C*_max_	−0.001420	0.0088	0.999	0.982	1.016	0.8720
	AUC_0–28day_	−0.000230	0.000202	1.000	0.999	1.000	0.2625
**Myocardial Injury** (*n* = 11)	*C*_max_	0.005440	0.005950	1.005	0.994	1.017	0.3605
	AUC_0–28day_	0.000027	0.000051	1.000	1.000	1.000	0.5978
**Possible ischemic related** (*n* = 7)	*C*_max_	0.006280	0.00684	1.006	0.993	1.020	0.3581
	AUC_0–28day_	0.000013	0.000075	1.000	1.000	1.000	0.8575
**Other myocardial injury** (*n* = 4)	*C*_max_	0.003340	0.0108	1.003	0.982	1.025	0.7580
	AUC_0–28day_	0.000041	0.000066	1.000	1.000	1.000	0.5301
**Miscellaneous Cardiac Event** (*n* = 7)	*C*_max_	−0.003290	0.0111	0.997	0.975	1.019	0.7667
	AUC_0–28day_	−0.000070	0.000155	1.000	1.000	1.000	0.6470

* Probability from a Wald Chi Square test. The control arm for all analyses was the population without cardiac event and with PK data (*n* = 375); AUC: area under the curve; CI: confidence interval; *C*_max_: maximum plasma concentration; OR: odds ratio; PK: pharmacokinetic; SE: standard error.
